# Descriptions of a new species of *Foenatopus* Smith from China and the male of *Parastephanellus brevicoxalis* (Hymenoptera, Stephanidae)

**DOI:** 10.3897/zookeys.612.9781

**Published:** 2016-08-23

**Authors:** Hua-yan Chen, Cornelis van Achterberg, Zai-fu Xu

**Affiliations:** 1 Department of Entomology, The Ohio State University, 1315 Kinnear Road, Columbus, Ohio 43212, U.S.A.; 2 Key Laboratory of Resource Biology and Biotechnology in Western China (Northwest University), Ministry of Education; School of Life Sciences, Northwest University, 229 North Taibai Road, Xi’an, Shaanxi 710069, China; 3 Department of Entomology, South China Agricultural University, Guangzhou 510640, P. R. China

**Keywords:** China, Foenatopus, male, new species, Oriental Region, Parastephanellus brevicoxalis

## Abstract

A new species of the stephanid genus *Foenatopus* Smith, *Foenatopus weii* **sp. n.**, is described and illustrated from Yunnan Province, China. A modified section of the identification key to species of *Foenatopus* is added to include the new species. The male of *Parastephanellus brevicoxalis* Hong, van Achterberg & Xu, 2011 from Guangdong Province, China is also described and illustrated for the first time.

## Introduction

The family Stephanidae Leach, 1815 are a rare group of parasitoids (Hong et al. 2011). The Chinese Stephanidae were recently revised by Hong et al. (2011), and five genera and 21 species were recognized. However, one species of *Schlettererius* Ashmead, 1900 (Tan et al. 2015) and two species of *Pseudomegischus* van Achterberg, 2002 (Tan et al. 2015; Chen et al. 2016) were subsequently reported from China, suggesting that the actual number of Stephanidae occurring in China is still underestimated. During recent surveys of Chinese Hymenoptera some additional specimens of Stephanidae have been collected. Among them, a new species of *Foenatopus* is recognized and the male of *Parastephanellus brevicoxalis* Hong, van Achterberg & Xu, 2011 is reported for the first time. Here the new species *Foenatopus weii* sp. n. and the male of *Parastephanellus brevicoxalis* Hong, van Achterberg & Xu, 2011 are described.

## Material and methods

Descriptions of the species have been made under an Olympus SZ61 stereomicroscope, with lighting achieved through a 27W fluorescent lamp. Digital images were taken with a digital microscope KEYENCE® VHX-5000 (Osaka, Japan), and plates were edited with the programs ACDSee 10.0 and Photoshop CS 8.0.1.

Morphological nomenclature follows van Achterberg (2002) and Hong et al. (2011).

The female holotype of *Parastephanellus brevicoxalis* Hong, van Achterberg & Xu, 2011 is deposited in Zhejiang University (ZJUH). The remaining examined material is deposited in the Hymenopteran Collection, South China Agricultural Universtiy, Guangzhou, China (SCAU).

## Taxonomy

### Foenatopus

Taxon classificationAnimaliaHymenopteraStephanidae

Smith, 1861

Foenatopus Smith 1861: 58. Type species (by monotypy): Stephanus indicus Westwood, 1841.

#### Notes.

The Chinese *Foenatopus* were recently revised by Hong et al. (2011). Eleven species of the genus are known from China after this study.

### Foenatopus weii sp. n.

Taxon classificationAnimaliaHymenopteraStephanidae

http://zoobank.org/261245ED-87D0-4FB7-B110-09EBBBC398A7

Figs 1 , 2–5 , 6–11

#### Material examined.

Holotype, ♀ (SCAU), CHINA: Yunnan, Jinghong, Nanbanhe National Nature Reserve, 22°15'47.39"N, 100°36'3.22"E, 892 m, 19–23.VII.2011, Nasen Wei, yellow pan trap.

#### Etymology.

Named after the collector Dr. Nasen Wei.

#### Diagnosis.

Frons finely and transversely carinate-rugose (Fig. 2); vertex finely and transversely striate (Fig. 4); pronotum slender and mostly coriaceous, but anteriorly transversely rugulose (Figs 3, 5); scutellum (Fig. 5), propleuron and mesopleuron (Figs 3, 6) coriaceous; propodeum (Figs 5, 6) with medium-sized, circular foveolae and with coriaceous interspaces, inside of foveolae polished; fore wing with vein 2-CU1 absent (Fig. 9); most of mid tarsus ivory; subapical part of ovipositor sheath whitish (Fig. 11). This species is most similar to *Foenatopus flavidentatus* (Enderlein, 1913) but can be distinguished by the combination of the following characteristics: frons with two rather than three short longitudinal ivory streaks; pronotum entirely coriaceous (smooth posteriorly in *Foenatopus flavidentatus*); propodeum more spaced foveolate (reticulate-foveolate in *Foenatopus flavidentatus*); hind femur entirely black (hind femur chestnut brown and with two large ventral teeth yellowish in *Foenatopus flavidentatus*).

**Figure 1.**
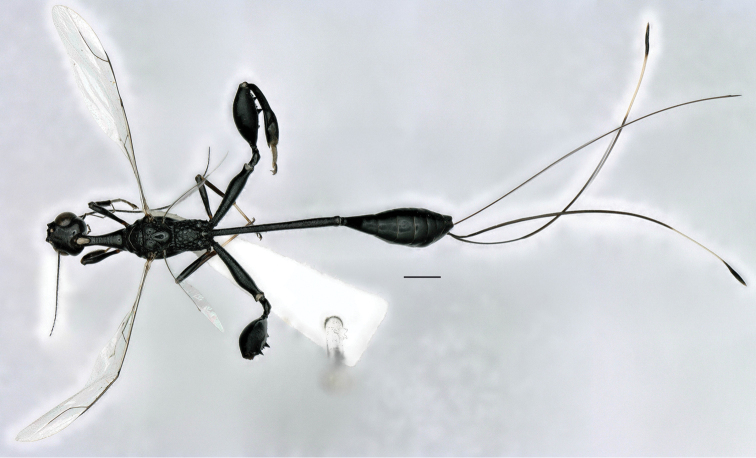
*Foenatopus weii* sp. n., holotype, female, dorsal habitus. Scale bar: 1 mm.

**Figures 2–5.**
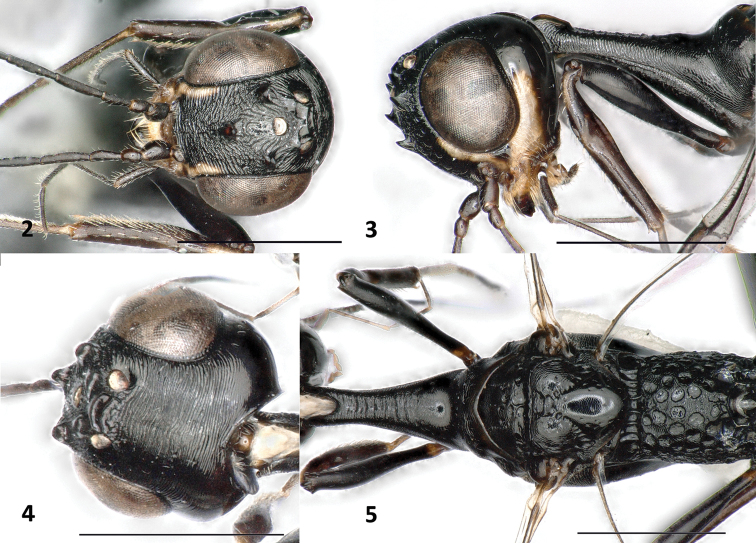
*Foenatopus weii* sp. n., holotype, female. **2** Head frontal **3** head and pronotum lateral **4** head dorsal **5** mesosoma dorsal. Scale bars: 1 mm.

**Figures 6–11.**
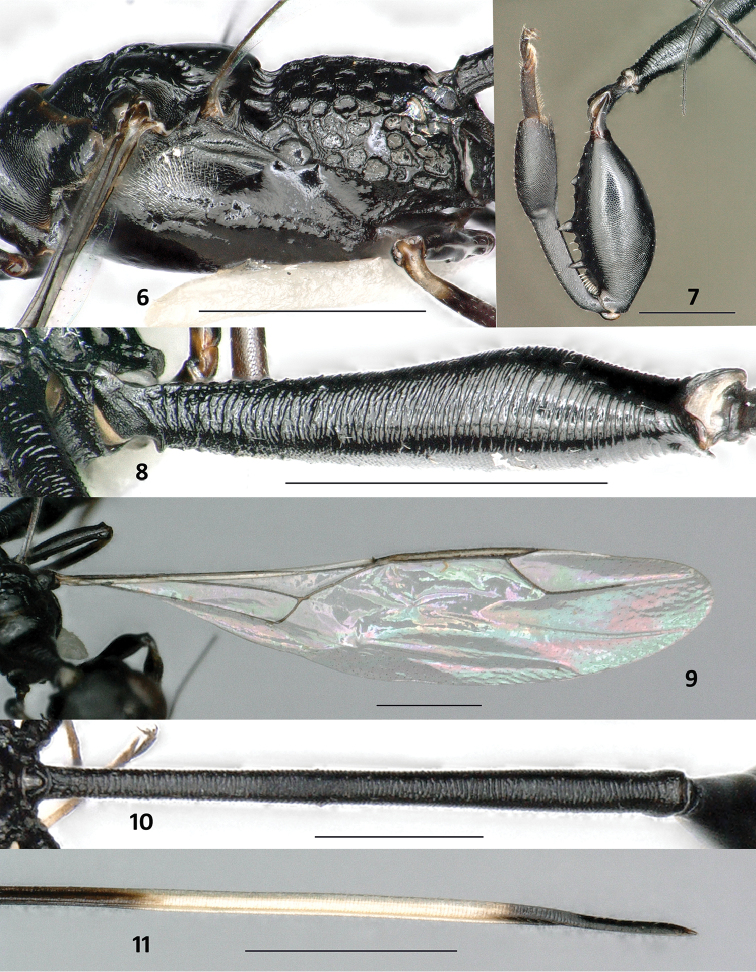
*Foenatopus weii* sp. n., holotype, female. **6** Mesopleuron, metapleuron and propodeum lateral **7** hind leg **8** hind coxa lateral **9** fore wing **10** first tergite dorsal **11** apical ovipositor sheath. Scale bars: 1 mm.

#### Description.

Holotype. *Female*. Body length 12.2 mm; fore wing length 6.1 mm.

*Colour*. Black (Fig. 1), except: frons laterally with short ivory streaks not reaching level of anterior coronal tooth (Fig. 2); temple brown with ivory streak along lower half of the eye, streak becoming narrow apically (Fig. 3); base of mandible yellow; most of mid tarsus ivory; subapical part of ovipositor sheath whitish (Fig. 11).

*Head*. Antenna with 33 segments; first flagellomere 3.0 × as long as wide, and 0.6 × as long as second flagellomere; three anterior coronal teeth large and acute, both posterior ones short and wider; frons finely and transversely carinate-rugose (Fig. 2); coronal area with some longitudinal carinae; vertex finely and transversely striate (Fig. 4); temple non-angulate, smooth and shiny (Fig. 3).

*Mesosoma*. Pronotum (Figs 3, 5) slender and mostly coriaceous, but with transverse rugulae anteriorly; neck anteriorly deeply emarginated, neck at slightly lower level than middle part of pronotum (Fig. 3); anterior half of mesoscutum transversely coriaceous, posterior half with shallow and large foveolae; notauli and middle groove distinct; scutellum (Fig. 5), propleuron and mesopleuron (Figs 3, 6) coriaceous; propodeum (Figs 5, 6) with medium-sized, circular foveolae and with wide coriaceous interspaces, inside of foveolae polished; fore wing (Fig. 9) with vein 2-CU1 absent; pterostigma elongate and subparallel-sided, acute apically, 15.4 × as long as its maximum width and 3.1 × as long as vein r; vein r and vein SR1 obtusely-angled, vein r ends 0.2 × length of pterostigma behind level of apex of pterostigma; vein SR1 subparallel to costal margin; hind coxa transversely striate, basal third rugose; hind femur swollen, microreticulate, ventrally with two large acute teeth and with one smaller tubercle basally (Fig. 7); hind tibia coriaceous, 1.2 × as long as hind femur, basal narrow part of hind tibia 1.2 × as long as widened part, inner side of widened part basally distinctly depressed, followed by convex and setose area, apically densely setose.

*Metasoma*. First tergite transversely striate, 14.7 × as long as its maximum width, 3.5 × as long as second tergite and 1.1 × as long as remainder of metasoma; basal 0.1 of second tergite rugose, remaining tergites largely smooth to weakly coriaceous; pygidial area distinctly differentiated, pygidial impression reverse V-shaped; length of ovipositor sheath 0.7 × as long as body length, length of subapical whitish band 1.9 × length of dark apex (Fig. 11).

Male. Unknown.

#### Distribution.

Oriental: China (Yunnan).

#### Biology.

Collected in July. Host not known.

In the key to species of the genus *Foenatopus* by Hong et al. (2011), the new species can be included by replacing couplet 8 as follows:

**Table:** 

8	Middle pale stripe of frons comparatively wide dorsally (Figs 124, 133 in Hong et al. 2011) and base of anterior tooth of corona yellowish-brown; pronotum often yellowish-brown or dark brown posteriorly and usually contrasting with black mesoscutum (Figs 127, 151 l.c.); teeth of hind femur completely to partly pale yellowish or ivory (Fig. 129 l.c.)	***Foenatopus flavidentatus* (Enderlein, 1913)**
–	Middle pale stripe of frons absent (Fig. 2) or narrow dorsally (Fig. 76 l.c.) and base of anterior tooth of corona dark brown or black; pronotum black posteriorly and as black as mesoscutum (Figs 59, 78 l.c., also Fig. 5); teeth of hind femur often completely or largely black or dark brown (Figs 61, 71 l.c., also Fig. 7)	**9**
9	Pronotum with posterior half distinctly striate or carinate (Figs 175, 176 l.c.); face of female without distinct pale lateral stripes (Fig. 182 l.c.); frons comparatively coarsely sculptured (Fig. 182 l.c.)	***Foenatopus quadridens* (Elliott, 1920)**
–	Pronotum with posterior half mainly reticulate-coriaceous (Fig. 5), at most with some short striae or carinae (Figs 50, 59, 68, 78 l.c.); face of female with distinct pale lateral stripes (Fig. 2); frons comparatively finely sculptured (Fig. 2)	**10**
10	Middle stripe of frons abent (Fig. 2); anterior half of mesoscutum coriaceous (Fig. 5); propodeum (Figs 5, 6) with medium-sized, circular fovelae and with wide coriaceous interspaces	***Foenatopus weii* sp. n.**
–	Middle stripe of frons present (Fig. 2); anterior half of mesoscutum striate (Fig. 5); propodeum strongly and densely reticulate-foveolate, the foveolae rather large and irregularly shaped (Figs 52, 70 l.c.)	**11**
11	Ovipositor sheath completely black (Fig. 54 l.c.); anterior half of pronotum in lateral view without transverse carinae and flat medially or slightly impressed (Fig. 51 l.c.); fore wing with vein 2-CU1 absent (Fig. 49 l.c.)	***Foenatopus brevimaculatus* Hong, van Achterberg & Xu, 2011**
–	Ovipositor sheath with ivory subapical band (Figs 62, 73 l.c.); anterior half of pronotum in lateral view with transverse carinae and depressed medially (Fig. 79 l.c.); fore wing with vein 2-CU1 weakly developed, 0.2 × as long as cu-a (Fig. 67 l.c.)	***Foenatopus chinensis* (Elliott, 1919)**

### Parastephanellus

Taxon classificationAnimaliaHymenopteraStephanidae

Enderlein, 1906

Parastephanus Enderlein, 1905: 474 (not Haeckel 1881). Type species (by original designation): Stephanus pygmaeus Enderlein, 1901.
Parastephanellus Enderlein, 1906: 301. Type species (by original designation): Stephanus pygmaeus Enderlein, 1901.

#### Notes.

At present five species of *Parastephanellus* are known from China with four species are only described from females or males. Here the male of *Parastephanellus brevicoxalis* Hong, van Achterberg & Xu, 2011 is described.

### Parastephanellus brevicoxalis

Taxon classificationAnimaliaHymenopteraStephanidae

Hong, van Achterberg & Xu, 2011

Figs 12 , 13–15 , 16–21

Parastephanellus brevicoxalis Hong, van Achterberg & Xu 2011: 39.

#### Material examined.

Holotype, ♀ (ZJUH), CHINA: Zhejiang, Wuyanling Provincial Nature Reserve, 29. VII.2005, Peng Xu, No. 200605074. Other material. 1♂ (SCAU): CHINA: Guangdong, Nanling National Nature Reserve, 6.X.2004, Zaifu Xu.

#### Description.

*Male*. Body length 9.6 mm; fore wing length 5.3 mm.

**Figure 12.**
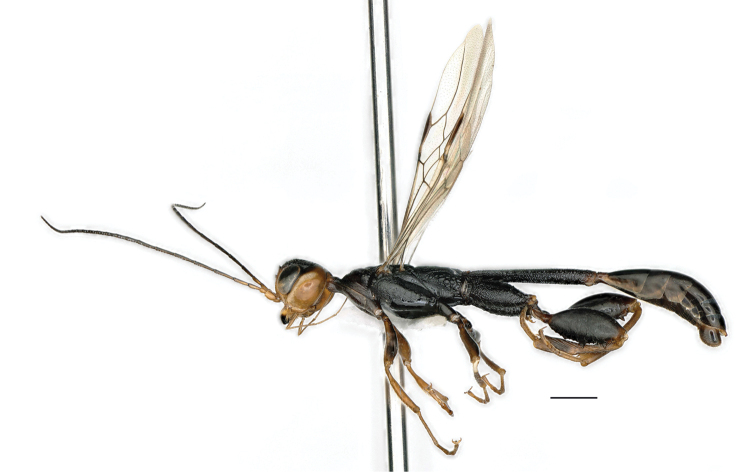
*Parastephanellus brevicoxalis* Hong, van Achterberg & Xu, 2011, male, dorsal habitus. Scale bar: 1 mm.

*Colour*. Black (Fig. 12), except: frons yellowish-brown; coronal teeth, vertex medio-longitudinally and narrow area of vertex behind eyes dark brown, remainder of vertex reddish-brown; temple yellowish-brown with yellow streaks along eye; base of mandible yellow; palpi, scape, pedicel yellowish-brown; propleuron largely dark brown; fore leg, tibiae and tarsi of mid and hind legs yellowish-brown; hind trochanter and base of second tergite reddish-brown; wing membrane subhyaline; pterostigma and wing venation dark brown.

**Figures 13–15.**
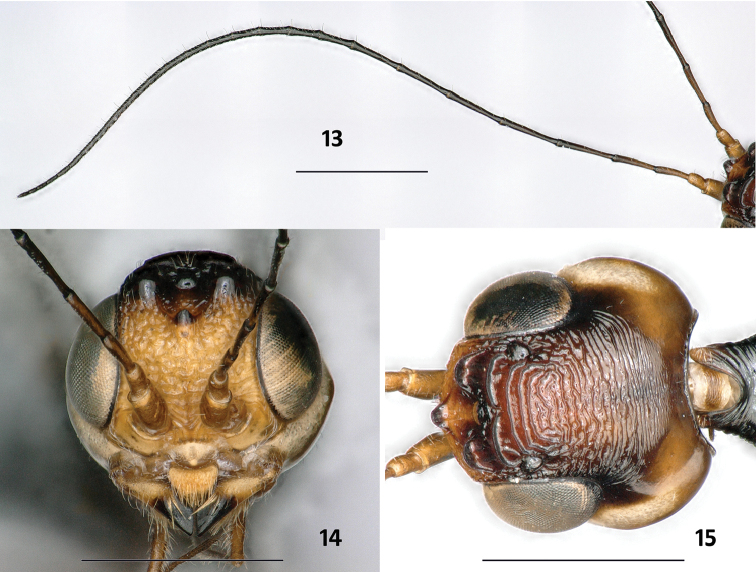
*Parastephanellus brevicoxalis* Hong, van Achterberg & Xu, 2011, male. **13** Antenna **14** head frontal **15** head dorsal. Scale bars: 1 mm.

*Head*. Antenna with 28 segments (Fig. 13); frons coarsely reticulate-rugose (Fig. 14); three anterior coronal teeth acute, both posterior ones wide and arcuate, sculpture on coronal area from rugose anteriorly to longitudinally short carinate; behind level of coronal area with three strong, transverse straight carinae, followed by transversely rugose area, rugae coarse anteriorly, finer laterally near eye and posteriorly, striae posteriorly weaker and approximately extending to occipital carina (Fig. 15); temple smooth and shiny, relatively broad; gena round (Fig. 15).

**Figures 16–21.**
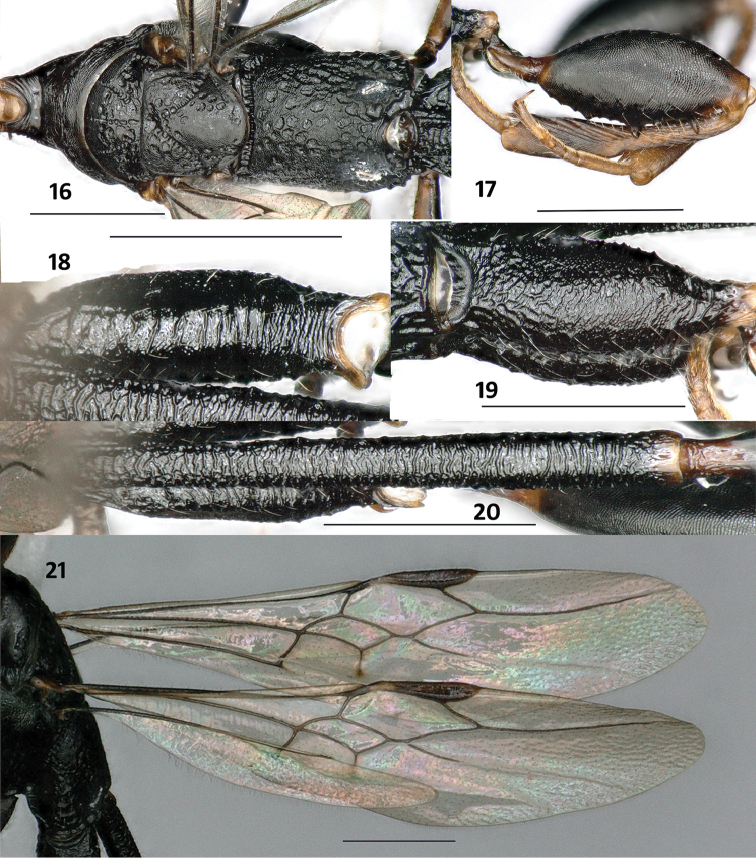
*Parastephanellus brevicoxalis* Hong, van Achterberg & Xu, 2011, male. **16** Mesosoma dorsal **17** hind femur and tibia lateral **18** hind coxa dorsal **19** hind coxa lateral **20** first tergite dorsal **21** wings. Scale bars: 1 mm.

*Mesosoma*. Neck (Fig. 16) short and robust, anteriorly distinctly emarginate, medio-posteriorly smooth, and with pairs of oblique lateral carinae, neck at much lower level than remainder of pronotum; pronotal fold and concavity absent; middle of pronotum steeply elevated and subvertical to neck, weakly transversely striate; posterior pronotum not differentiated from middle part, weakly striate dorsally and more or less smooth apically (Fig. 16), pronotal lobe with oblique striae; lateral oblique groove of pronotum narrow and indistinct, ventral area below it distinctly obliquely striate (Fig. 16); propleuron largely coriaceous, smooth medially; mesoscutum foveolate, anterior 0.2 and area between foveolae striate, latero-posteriorly somewhat rugose; notauli and median groove distinct and formed by some foveolae or crenulae; axilla rugose-foveolate; scutellum (Fig. 16) laterally densely foveolate and medially rugulose; mesopleuron rather robust, dorsally flat and largely smooth, convex ventral part shallowly rugose and pubescent, anteriorly pubescence denser and rugae coarser than posteriorly; convex part of metapleuron irregularly rugose and sparsely setose, ventral part below it rugulose; propodeum irregularly foveolate, foveolae changing from circular to suboval, area in between and inside foveolae rugulose, foveolae laterally and apically somewhat coalescent and reticulate (Fig. 16); fore wing (Fig. 21) with vein 1-M 1.9 × as long as vein 1-SR and 1.2 × vein m-cu; vein cu-a slightly postfurcal and subvertical; vein 2-SR 1.7 × as long as vein r; vein r ends at level of apex of pterostigma; vein r and vein 1-M distinctly curved; vein 1-SR 1.4 × as long as parastigmal vein; vein 3-CU1 basal 0.2 tubular, remainder largely nebulous, apically distinctly curved; hind coxa (Figs 17–19) robust, antero-dorsally rugose, anterior 0.6 of outer side distinctly compressed and sculpture changing from rugose to microreticulate, posterior part of hind coxa coarsely transversely striate; hind femur (Fig. 17) considerably swollen, densely and finely aciculate, ventrally with 2 large teeth and some denticles in between; hind tibia (Fig. 17) about 1.2 × as long as hind femur, basal narrow part about 1.4 × as long as widened part, outer side of hind tibia distinctly obliquely carinate, narrow part of inner side coriaceous, widened part of inner side distinctly depressed basally and densely bristly setose apically; basitarsus rather robust, ventral length about 3.8 × as long as its apical width.

*Metasoma*. First tergite 7.5 × as long as its maximum width, 2.6 × as long as second tergite and 0.8 × as long as remainder of metasoma, densely coarsely and rather regularly transversely striate, basal 0.1 rugose and with 2 distinct, short longitudinal carinae, apically narrowly smooth; basal 0.2 of second tergite with several short longitudinal carinae, remainder of tergite smooth; remainder of tergites densely finely microaciculate; pygidial process distinct and tubular apically.

#### Distribution.

Oriental: China (Zhejiang, Guangdong).

#### Biology.

Collected in July and October. Host not known.

#### Remark.

The male is similar to the female, except: body smaller (female body length 16.2 mm); head paler; antenna with 28 segments (33 segments in female); propleuron largely coriaceous, smooth medially (coriaceous and microreticulate in female); vein 1-M 1.9 × as long as vein 1-SR and 1.2 × as long as vein m-cu (vein 1-M 1.25 × as long as vein 1-SR and 0.9 × as long as vein m-cu in female); vein r ends at level of apex rather than behind of pterostigma; first tergite elongate, considerably longer than second tergite.

## Supplementary Material

XML Treatment for Foenatopus

XML Treatment for Foenatopus weii

XML Treatment for Parastephanellus

XML Treatment for Parastephanellus brevicoxalis
